# Update on SARS-CoV-2 Omicron Variant of Concern and Its Peculiar Mutational Profile

**DOI:** 10.1128/spectrum.02732-21

**Published:** 2022-03-30

**Authors:** Mohammad Alkhatib, Romina Salpini, Luca Carioti, Francesca Alessandra Ambrosio, Stefano D’Anna, Leonardo Duca, Giosuè Costa, Maria Concetta Bellocchi, Lorenzo Piermatteo, Anna Artese, Maria Mercedes Santoro, Stefano Alcaro, Valentina Svicher, Francesca Ceccherini-Silberstein

**Affiliations:** a Department of Experimental Medicine, University of Rome Tor Vergata, Rome, Italy; b Dipartimento di Scienze della Salute, Università degli Studi “Magna Graecia” di Catanzaro, Campus S. Venuta, Catanzaro, Italy; c Net4Science Academic Spin-Off, Università Magna Græcia di Catanzaro, Campus S. Venuta, Catanzaro, Italy; d Department of Biology, University of Rome Tor Vergata, Rome, Italy; University of Georgia

**Keywords:** B.1.1.519, COVID-19, emerging variants, mutations, omicron, pandemic, SARS-CoV-2, variant of concern

## Abstract

The process of severe acute respiratory syndrome coronavirus 2 (SARS-CoV-2) genetic diversification is still ongoing and has very recently led to the emergence of a new variant of concern (VOC), defined as Omicron or B.1.1.529. Omicron VOC is the most divergent variant identified so far and has generated immediate concern for its potential capability to increase SARS-CoV-2 transmissibility and, more worryingly, to escape therapeutic and vaccine-induced antibodies. Nevertheless, a clear definition of the Omicron VOC mutational spectrum is still missing. Herein, we provide a comprehensive definition and functional characterization (in terms of infectivity and/or antigenicity) of mutations characterizing the Omicron VOC. In particular, 887,475 SARS-CoV-2 Omicron VOC whole-genome sequences were retrieved from the GISAID database and used to precisely define its specific patterns of mutations across the different viral proteins. In addition, the functional characterization of Omicron VOC spike mutations was finely discussed according to published manuscripts. Lastly, residues characterizing the Omicron VOC and the previous four VOCs (Alpha, Beta, Gamma, and Delta) were mapped on the three-dimensional structure of the SARS-CoV-2 spike protein to assess their localization in the different spike domains. Overall, our study will assist with deciphering the Omicron VOC mutational profile and will shed more light on its clinical implications. This is critical considering that Omicron VOC is currently the predominant variant worldwide.

**IMPORTANCE** The Omicron variant of concern (VOC) has a peculiar spectrum of mutations characterized by the acquisition of mutations or deletions rarely detected in previously identified variants, particularly in the spike glycoprotein. Such mutations, mostly residing in the receptor-binding domain, could play a pivotal role in enhancing severe acute respiratory syndrome coronavirus 2 (SARS-CoV-2) infectivity (by increasing binding affinity for ACE2), jeopardizing spike recognition by therapeutic and vaccine-induced antibodies and causing diagnostic assay failure. To our knowledge, this is one of the first exhaustive descriptions of newly emerged mutations underlying the Omicron VOC and its biological and clinical implications.

## INTRODUCTION

On the 26 November 2021, the World Health Organization (WHO) has officially disclosed the emergence of a novel SARS-CoV-2 variant of concern (VOC) defined as Omicron (Pango Lineage B.1.1.529 and Nextstrain Clade 21K), only 48 h after its detection. This VOC is the most divergent SARS-CoV-2 variant evolved so far and thus has immediately raised concerns for its potential implications in terms of increased transmissibility and risk of reinfections, as well as reduced vaccine effectiveness (1–3).

The novel Omicron VOC was first detected in South Africa from a sample collected at the beginning of November 2021 and then in Botswana and Hong Kong; it later spread in dozens of countries, including several European countries and the United States (1–3). From an evolutionary point of view, the origin of the Omicron VOC is uncertain. Indeed, phylogenetic analyses have shown that this variant did not originate from one of the previously identified VOCs. Conversely, it appears to have evolved in parallel from an ancestor presumably developed in mid-2020 (4). Nonetheless, different hypotheses have been formulated on its origin, including the generation in nonhuman species and the evolution in an immune-suppressed individual with a long-term infection (4), while more recently, it has been postulated that Omicron could have evolved as a result of a recombination event with SARS-CoV-2 and common cold coronavirus E229 in coinfected patients (5). A recent study has shown the capability of Omicron VOC to replicate more efficiently in the upper than in the lower respiratory tract, due to a TMPRSS2 protease-independent mechanism of entry supporting its high transmissibility (6).

Based on epidemiological evidence, it has been estimated that the Omicron VOC is characterized by a 2- to 3-fold increased risk of reinfection, suggesting its capability to evade preexisting immunity (7). Interestingly, several of the latest studies have shown that the Omicron VOC determines to a different extent the reduction in antibody neutralization elicited by the two doses of the BNT162b2, ChAdOx1, or CoronaVac vaccines compared to the ancestral strain harboring the D614G mutation and to other preexistent variants such as Alpha and Delta (8–12). In particular, the results indicated that two doses of vaccination with BNT162b2, ChAdOx1, or CoronaVac are insufficient to give adequate levels of protection against infection and mild disease sustained by the Omicron VOC at any time points analyzed post-second dose vaccination (10–14). Luckily, there is evidence that booster doses of homologous or heterologous BNT162b2 provide a significant increase in protection against mild (and presumably) severe disease, supporting the need to maximize coverage with third doses of vaccine (10, 12–14). In particular, preliminary laboratory data from Pfizer showed that the third dose determines a 25-fold increase in the titer of neutralizing antibodies (14). Sera obtained 1 month after receiving booster dose can neutralize the Omicron VOC, although at levels lower than those observed with the wild-type virus and with other variants (9–11). In a similar direction, several studies have demonstrated the retainment of considerable immunity in individuals who have been infected prior to being fully vaccinated (8, 10, 12, 14).

**FIG 1:**
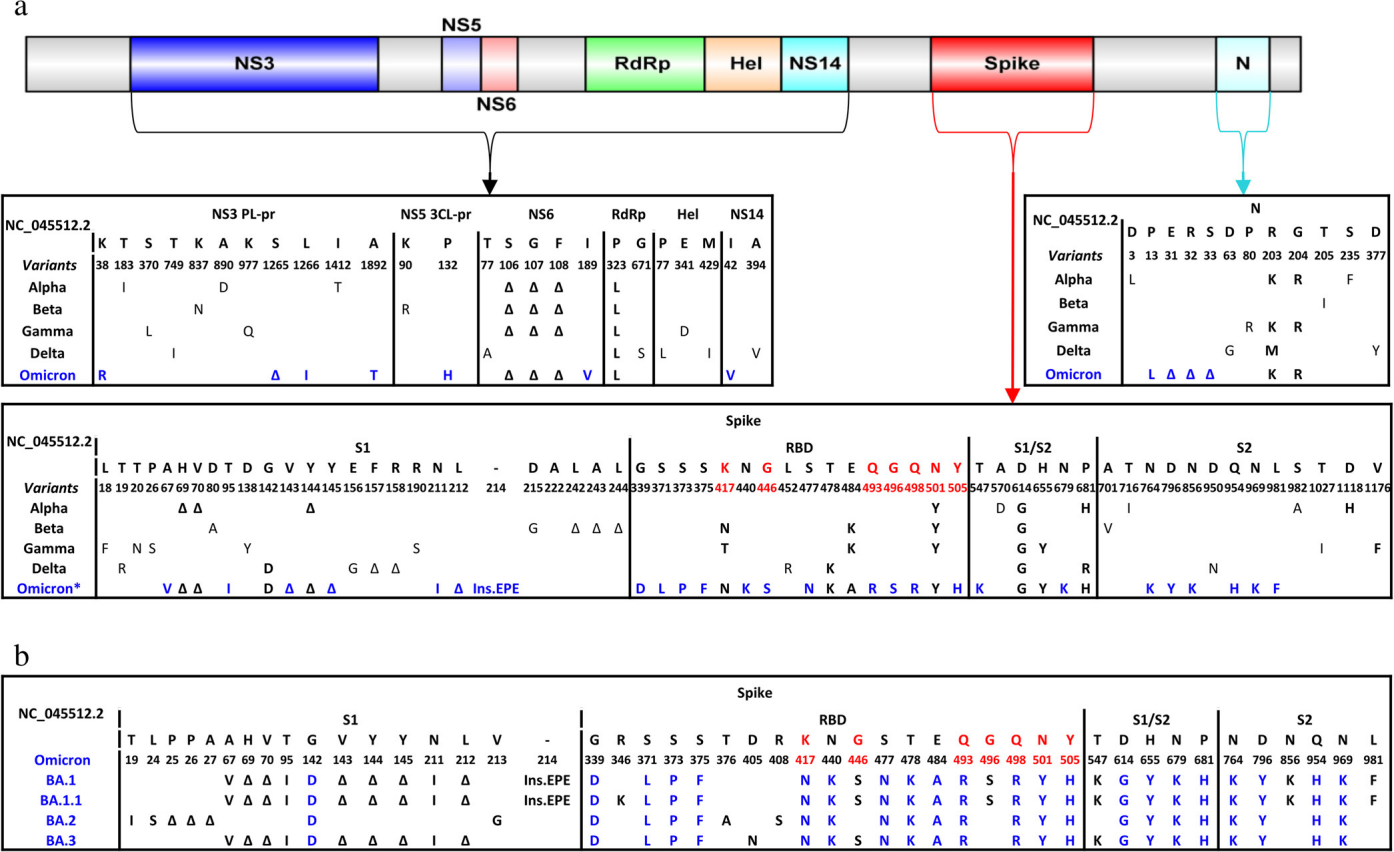
(a) Schematic illustration of severe acute respiratory syndrome coronavirus 2 (SARS-CoV-2) genome and its variants of concern along with their relevant mutations that characterize each protein of interest. Mutations were defined as amino acid substitutions/deletions/insertions that occurred in 75% of sequences using the NC_045512.2 as the reference sequence. Residues in red are those that directly interact with ACE2. Mutations in bold black refer to the shared mutations by at least two variants of concern, while those in bold blue refer to the unique Omicron mutations. *Mutations at residues 417, 440, and 446 in the receptor-binding domain (RBD) spike represent original Omicron consensus according to earlier prevalence of greater than 75%, while currently, the new prevalence values are 51.3, 53.9, and 54,9%, respectively. (b) Spike mutations underlying the currently circulating Omicron sublineages BA.1, BA.1.1, BA.2, and BA.3. Blue color refers to those mutations shared by the four sublineages.

The Omicron VOC has a peculiar mutational profile characterized by an extremely high number of mutations (N = 65, including 16 deletions and 3 insertions) compared to previously identified variants. Most of them localize in the spike glycoprotein, a key mediator of viral infectivity and the protein primarily targeted by vaccines and monoclonal and polyclonal antibodies. Furthermore, the Omicron VOC is already undergoing a process of further genetic evolution as attested by the recent identification of four sublineages according to Pango Lineage (BA.1, BA.2, BA.3, and BA.1.1). The B.1.1.529 Omicron VOC ancestor has been designated BA.1. Interestingly, the novel BA.2 has undergone a more pronounced genetic rearrangement characterized by the accumulation (T19I, L24S, P25-P26-A27del, V213G, T376A, D405N, and R408S) or loss (V67A, H69-V70del, T95I, V143-Y144-Y145del, N211I L212del, Ins214EPE, G446S, G496S, T547K, N856K, and L981F) of several mutations (Fig. 1). Conversely, BA.3 shared the entire mutational set with BA.1, with the addition of the receptor-binding domain (RBD) mutation D405N and the loss of the Ins214EPE, G496S, N856K, and L981F, while the BA.1.1 has added only a single RBD mutation, R346K, and kept BA.1’s entire mutational set (Fig. 1). A very recent Danish study has demonstrated that Omicron BA.2 sublineage was linked with higher susceptibility of infection than the original Omicron BA.1, regardless of vaccination status (15).

Based on these assumptions, there is an urgent need to provide a comprehensive definition of the mutational patterns (in the full-length SARS-CoV-2 genome) characterizing the Omicron VOC. Thus, this is one of the first extensive definitions and characterizations of the mutational profile underlying the Omicron VOC and its biological and clinical implications.

## RESULTS AND DISCUSSION

### Spike mutational profiles characterizing Omicron VOC.

**FIG 2:**
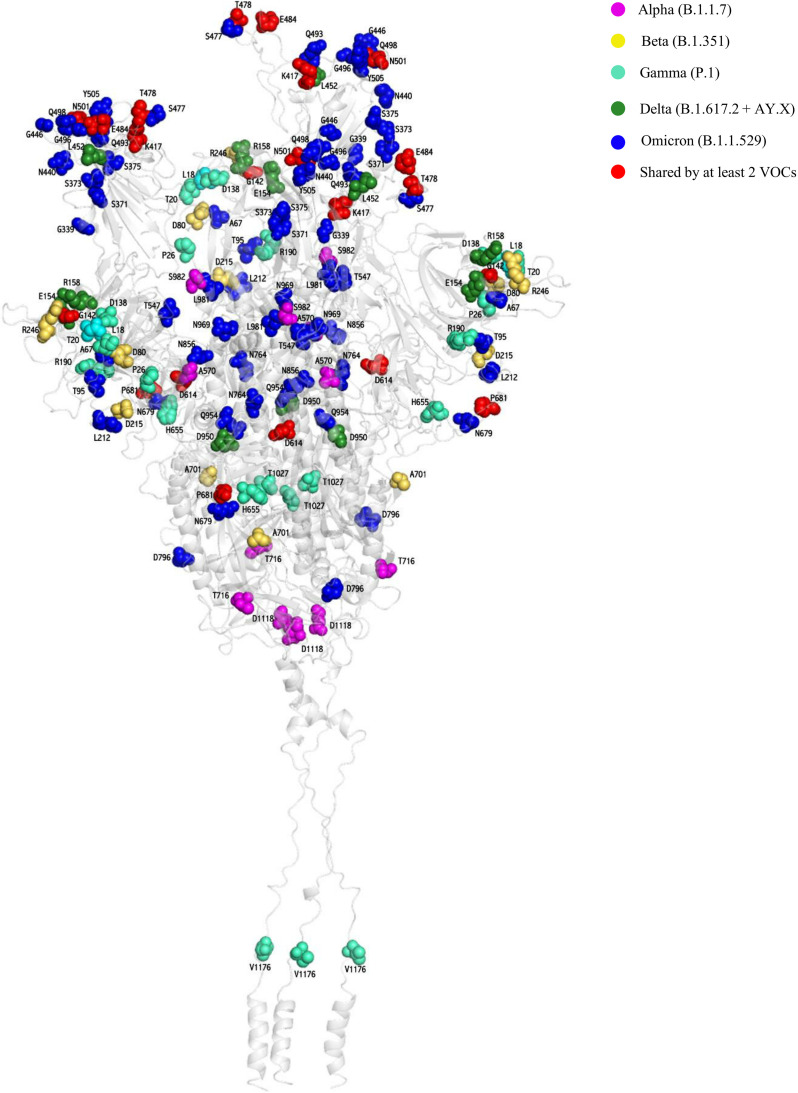
Three-dimensional representation of SARS-CoV-2 spike protein reporting residues characterizing the five variants of concern (VOCs). The protein is shown as a gray cartoon. The Alpha B.1.1.7, Beta B.1.351, Gamma P.1, Delta B.1.617.2 + AY.X, and Omicron B.1.1.529 VOCs are represented as magenta, yellow, cyan, forest green, and blue spheres, respectively. The shared mutated residues present in at least two VOCs are reported as red spheres.

The Omicron VOC is characterized by 39 mutations in the spike glycoprotein. Among them, 15 reside within the RBD, particularly in the receptor-binding motif (N = 10) that interacts directly with the ACE2 receptor and contains several neutralizing epitopes (Fig. 1; Table 1). Furthermore, 13 mutations, including 6 amino acid deletions (H69-V70del, V143-Y144-Y145del, and L212del) and 3 amino acid insertions (EPE214ins), have been identified in the N-terminal domain (NTD), known to contain specific epitopes highly targeted by monoclonal antibodies (16). The remaining 11 mutations are localized at the S1/S2 junction (fusion domain) and S2 subunit (Fig. 1). Notably, 27 of 39 mutations were never or rarely detected in the other SARS-CoV-2 variants, while the remaining are shared with the other VOCs: 6 with Alpha, 5 with Gamma, and 4 with both Beta and Delta (Table 1; Fig. 1 and 2).

**TABLE 1:** Spike mutations present in the Omicron variant of concern and their functional characterization^*a*^

Mutation	Variant	Location	Potential impact						
			Increased infectivity^*b*^	Increased transmissibility^*c*^	Increased disease severity^*d*^	Monoclonal and polyclonal antibody escape	Convalescent sera escape	Vaccine escape	Diagnostic assays escape
Amino acid mutations characterizing SARS-CoV-2 variants									
A67V	Omicron	NTD	NA	NA	NA	NA	NA	NA	NA
T95I	Omicron	NTD	No	No	No	NA	NA	NA	NA
G142D^*e*^	Omicron, Delta	NTD	NA	NA	NA	Yes	NA	NA	NA
L212I	Omicron	NTD	NA	NA	NA	NA	NA	NA	NA
G339D	Omicron	RBD	Yes	NA	NA	Yes	NA	NA	NA
S371L	Omicron	RBD	NA	NA	NA	Yes	NA	NA	NA
S373P	Omicron	RBD	NA	NA	NA	Yes	NA	NA	NA
S375F	Omicron	RBD	NA	NA	NA	Yes	NA	NA	NA
K417N^*e*^	Beta, Omicron	RBD	No	No	No	Yes	Yes	Yes	No
N440K	Omicron	RBD	Yes	NA	NA	Yes	Yes	Yes	NA
G446S	Omicron	RBD	NA	NA	NA	Yes	NA	NA	NA
S477N^*e*^	Omicron	RBD	Yes	Yes	No	Yes	Yes	Yes	No
T478K^*e*^	Delta, Omicron	RBD	NA	NA	NA	Yes	NA	NA	NA
E484A	Omicron	RBD	NA	NA	NA	Yes	NA	NA	NA
Q493R	Omicron	RBD	Yes	NA	NA	Yes	NA	NA	NA
G496S	Omicron	RBD	NA	NA	NA	Yes	NA	NA	NA
Q498R	Omicron	RBD	Yes	NA	NA	Yes	NA	NA	NA
N501Y^*e*^	Alpha, Beta, Gamma, Omicron	RBD	Yes	Yes	No	Yes	Yes	Yes	No
Y505H	Omicron	RBD	NA	NA	NA	Yes	NA	NA	NA
T547K	Omicron	S1/S2	NA	NA	NA	NA	NA	NA	NA
D614G^*e*^	All variants	S1/S2	Yes	Yes	No	No	No	No	No
H655Y^*e*^	Gamma, Omicron	S1/S2	Yes	NA	NA	No	No	No	No
N679K	Omicron	S1/S2	Yes	NA	NA	No	No	No	No
P681H^*e*^	Alpha, Omicron	S1/S2	Yes	NA	No	No	No	No	No
Deletions and insertions characterizing SARS-CoV-2 variants									
Deletion of H69-V70^*e*^	Alpha, Omicron	NTD	Yes	No	No	Yes	No	No	Yes
Deletion of V143-Y144-Y145^*e*^	Alpha, Omicron	NTD	No	No	No	Yes	No	No	No
Deletion of N211	Omicron	NTD	NA	NA	NA	NA	NA	NA	NA
Insertion of 214 EPE	Omicron	NTD	NA	NA	NA	NA	NA	NA	NA

a The nomenclature of variants is that reported by the World Health Organization (WHO). The Omicron S2 mutations (N764K, D796Y, N856K, Q954H, N969K, and L981F) have not been reported in the table since no data are yet available on their functional characterization. NA, not available; NTD, N-terminal domain (amino acids [aa] 13 to 305); RBD, receptor-binding domain (aa 319 to 541); SARS-CoV-2, severe acute respiratory syndrome coronavirus 2; S1/S2, the junction between subunit S1 and S2 (aa 542 to 690).

b Infectivity was evaluated in pseudotyped viruses and/or by structural analysis from studies published on PubMed or bioRxiv.

c Transmissibility was evaluated by molecular epidemiology-based studies and/or *in vivo* studies published on PubMed or bioRxiv.

d Disease severity was evaluated by analyzing clinical outcomes in term of long-lasting infections and/or hospitalization period.

e These mutations are also present in other identified VOCs, with the exception of S477N, which was detected in the variants B.1.620 and B.1.160. The role of these mutations has been extensively discussed in our recently published article (14).

The known mutations (H69-V70del, V143-Y144-Y145del, K417N, S477N, T478K, N501Y, D614G, H655Y, and P681H) shared with the four VOCs and other variants have been extensively reviewed in our paper recently published in Microbiology Spectrum (17). Herein, we specifically focus on the functional characterization of newly emerged Omicron spike mutations or those whose role has been only recently better clarified.

### Newly emerged spike RBD mutations characterizing the Omicron VOC.

The Omicron VOC is characterized by a peculiar enrichment of mutations in the RDB (amino acids [aa] 319 to 541), that were rarely detected in previous variants (Fig. 1 and 2). In particular, 11 newly emerged mutations have been detected whose functional characterization is reported as follows.

Before Omicron identification, G339D was a rare mutation not exceeding the frequency of 0.1% in the analysis of SARS-CoV-2 sequences from the GISAID database. It has been associated with a modest increase in binding affinity with ACE2 (18) and a slight reduction (1.2-fold) in binding affinity with the sotrovimab monoclonal antibody (19). Moreover, a recent study has shown that G339D is associated with escape from a subset of neutralizing antibodies (20).

Similarly, S371L, S373P, and S375F were extremely rare mutations with prevalence never exceeding 0.09%. For this reason, a paucity of information is available on their impact in modulating viral infectivity and antigenicity. These mutations have been shown to potentially confer resistance to several neutralization antibodies (20–22). In particular, S371L broadly affected neutralization by monoclonal antibodies targeting different RBD epitopes (21). Additionally, a study has shown that S375F is associated with escape from only a specific subset of neutralizing antibodies (20), while another study reported the capability of S373P to reduce affinity for the CR3022, a monoclonal antibody that disrupts the spike protein homotrimerization interface (22).

N440K was an uncommon mutation detected initially in pangolin and bat samples collected in 2017 in China and so far found in several global lineages of SARS-CoV-2 with an overall prevalence of 0.3%. The acquisition of the positively charged Lys (K) has been associated with a 10- to 100-fold increase in infectious titer compared to viral strains with or without the D614G mutation (23), supporting its capability to enhance the production of mature viral particles. In keeping with this finding, from an epidemiological point of view, N440K has been reported in several clusters in India and has been proposed to be responsible for reinfection cases and rapid spread of SARS-CoV-2 in certain geographic areas (23–25).

Notably, N440K has been reported to confer high resistance to the monoclonal antibodies imdevimab and C135 only when administered alone, reinforcing the importance of using a combination of monoclonal antibodies to fully and efficiently neutralize the virus (21, 26–29). More recently, N440K has been reported to be associated with escape from several neutralizing antibodies, including those generated by vaccines (20).

G446S was another rare mutation, present with a frequency of less than 0.09% before Omicron emergence. This mutation is located in the tip of the RBD and has a direct interaction with ACE2; however, there is no further information about its impact on ACE2 binding affinity. Studies have shown that this mutation is associated with the reduced neutralizing activity of several monoclonal antibodies, including imdevimab and cilgavimab (20, 27, 30). This result can be explained by the steric hindrance imposed by this mutation as reported in a structure-based study (31).

E484A was a rare mutation (prevalence, 0.2%) detected since the beginning of the pandemic whose frequency has progressively increased particularly in combination with F486I. Mutations at position E484 (particularly E484K/Q, but also including E484A) are well-known to confer resistance to several monoclonal antibodies, such as bamlanivimab and casirivimab (20, 27, 30, 32, 33). Notably, a study highlighted the emergence of E484A (along with other mutations) in an immunosuppressed patient with a long-lasting infection, highlighting the role of suboptimal immunological pressure in driving the selection of mutations with immune-evasion activity (34).

Q493R was an extremely rare mutation with prevalence never exceeding 0.08% prior to Omicron emergence. This position has a direct interaction with ACE2 and has been shown to increase binding affinity to ACE2 (35). Notably, the acquisition of the positively charged Arg (R) is known to be critical for antibody binding. Furthermore, a study has shown that Q493R can create steric hindrance for the antibodies binding targeting RBD (31). These findings can explain why this mutation is associated with a reduced neutralization to bamlanivimab by more than 6,000-fold, to etesevimab by 232-fold, and worryingly also to the combination of both drugs by more than 100-fold (20, 32, 35) and to be resistant to C144 and to class 2 antibodies C627, C602, and C671 (26, 27).

G496S and Q498R were previously detected with a frequency of less than 0.09%. These residues have direct interactions with ACE2, suggesting the potential capability to enhance viral infectivity. Indeed, a study has shown that Q498R in combination with N501Y determines 4- and 50-fold increases in ACE2 binding affinity compared to N501Y alone and wild-type spike, respectively (36). Interestingly, such an effect was not observed when Q498R was present alone (36), supporting the synergistic role of mutations in modulating viral fitness. This epistatic effect between Q498R and N501Y can be explained by the fact that the presence of both mutations can favor the establishment of new interactions with multiple residues in ACE2 (Y501 with ACE2 residue 41, and R498 with ACE2 residues 42 and 36), forming a strong network of new interactions responsible for the increased binding affinity between RBD and ACE2 (36). This was confirmed by an *in vitro* study showing that the coevolution of Q498R and N501Y is associated with increased infectivity (36). Furthermore, G496S was associated with escape from a wide range of neutralizing antibodies (20), presumably explained by the steric interference exerted by this mutation as recently proposed by a structure-based study (31), while the Q498R was linked to reduce binding affinity to a subset of neutralizing antibodies (20).

Y505H was an extremely rare mutation with prevalence never exceeding 0.08% prior to Omicron emergence, and thus little information is available about its implications. Only one study has shown that Y505H is associated with escape from the monoclonal antibody casirivimab (20).

### Implications of mutations characterizing the Omicron VOC in modulating viral host spectrum.

It is noteworthy that some Omicron mutations reside at spike positions that have been shown to play an important role in modulating the viral host spectrum, paving the way for events of spillover across animal species. In this regard, a recent study has shown that the acquisition of positively charged amino acids at positions 493 and 498 (Q493K and Q498H) enables SARS-CoV-2 to infect mice by establishing interactions between the RBD and the murine ACE2 (37, 38). Similarly, the Omicron mutations Q493R and Q498R (again implying the acquisition of positively charged amino acid) were found to be selected after 30 passages in mouse lungs (sequence deposited in GISAID database with the accession number EPI_ISL_1666328 [39]), thus reflecting adaption of SARS-CoV-2 in mice.

In line with this finding, the Omicron mutation Y505H was detected during the early phase of the pandemic in March 2020 at the Bronx Zoo in the United States in respiratory secretions/feces samples collected from lions and tigers infected by SARS-CoV-2, thus being reported as the first case of a spillback event in nondomestic species (40) (GISAID accession number EPI_ISL_566038 and EPI_ISL_566037) (39). This was further confirmed in another study highlighting the critical role of mutations at position 505 in enlarging the viral host spectrum (38).

Overall findings support SARS-COV-2 potential to give origin to spillover events into and back from humans, enlarging its animal reservoir and fueling its genetic diversification. In this regard, a recent study has shown that Omicron spike can mediate enhanced entry into cells expressing several different animal ACE2s, including various domestic avian species, horseshoe bats, and mice, suggesting an increased propensity for reverse zoonosis (6). The role of Omicron VOC in this peculiar phenomenon deserves further investigation.

### Other Omicron spike mutations.

**(i) In N-terminal domain (amino acids 13 to 305).** The Omicron VOC is the only VOC characterized by the concomitant presence of three deletions in the NTD: H69-V70del, V143-Y144-Y145del, and L212del. H69-V70del and V143-Y144-Y145del were previously detected in other VOCs and variants of interest (VOIs) with an overall prevalence of more than 17% for both deletions, while L212del is a new deletion and specific for Omicron with a prevalence of less than 0.07% since the beginning of the pandemic. In particular, the H69-V70del was first detected in the Alpha VOC and is well-known to increase SARS-CoV-2 infectivity and to lead to S gene target failure by some molecular assays (41, 42).

It has been postulated that the above-mentioned deletions have independently emerged as a consequence of convergent evolution in immunocompromised individuals with long-lasting infection and have been proposed as a mechanism driving to an accelerated SARS-CoV-2 adaptive evolution and antigenic novelty since the RdRp proofreading activity cannot correct deletions (16). In this regard, V143-Y144-Y145del can confer resistance to some NTD-binding monoclonal antibodies (16). Notably, such resistance is further increased when V143-Y144-Y145del is copresent with other deletions, particularly with H69-V70del (16), further reinforcing their capability to favor SARS-CoV-2 evasion from antibodies targeting the NTD, particularly when combined. Further studies are urgently needed to unravel the immune-evasion potential associated with the copresence of these three deletions characterizing the Omicron VOC.

Beyond deletions, Omicron VOC is characterized by the insertion of three amino acids (E-P-E) at residue 214 with a prevalence of less than 0.07% since the beginning of the pandemic. Notably, a recent study has shown that this insertion is present also in seasonal coronaviruses HCoV-229E, thus leading to the hypothesis that it could have been acquired by template switching involving the genomes of HCoV-229E and SARS-CoV-2 in coinfected patients (5). Notably, a recent structure-based study has speculated that this insertion was not related to immune escape due to lack of overlap with known immune epitopes (43). No information on the functional role of this insertion is known so far.

Furthermore, the Omicron VOC is also characterized by the four point mutations in the NTD: A67V, T95I, G142D, and N211I with the prevalence of 0.6, 24.8, 40.9, and less than 0.003%, respectively (Fig. 1; Table 1). In particular, A67V was primarily detected in the Eta VOI, while T95I was described in the Delta VOC ancestor, Iota VOI, and other variants. According to recent literature, T95I can confer a significant advantage in terms of viral infectivity (44). Notably, G142D has been associated with an alteration of the supersite epitope that binds NTD-neutralizing antibodies by the *in silico* model (45). For N211I mutation, no information is available so far.

**(ii) In S1/S2 Junction (amino acids 542 to 690).** Several mutations were detected in the S1/S2 junction (T547K, D614G, H655Y, N679K, and P681H). These mutations had prevalence of 0.1, greater than 89.9, 2.2, 0.3, and 18.9%, respectively. Among them, H655Y and N679K are of interest since both reside close to the furin cleavage site (at S1/S2 aa 682 to 685). H655Y was previously detected in Gamma VOC and other variants and has been associated with increased spike cleavage and with a slight increase in ACE2 interaction, suggesting a role in enhancing viral infectivity (46, 47). This mutation was also selected after *in vivo* replication in the mink model, again suggesting its potential role in modulating the SARS-CoV-2 host spectrum (47). For T547K mutation, no information is available so far.

**(iii) In S2 subunit (amino acids 691 to 1273).** Several mutations were detected in the S2 domain of the Omicron VOC (N764K, D796Y, N856K, Q954H, N969K, and L981F). These mutations were rarely detected prior to Omicron VOC emergence with an overall prevalence of less than 0.04%. Although S2 has a critical role in mediating membrane fusion, the roles of mutations in this domain are poorly characterized.

### Omicron mutations localized in proteins other than the spike glycoprotein (N = 26).

Similar to the other variants, the Omicron VOC is characterized by several mutations in the other structural proteins localized in the viral envelope, such as T9I in the envelope protein, as well as D3G, Q19E, and A63T in the membrane protein. Notably, the Omicron VOC is also characterized by an enrichment of mutations in the nucleocapsid (P13L, R203K, and G204R) in addition to deletion of E31-R32-S33 (never detected before in other variants). This deletion and others in the nucleocapsid have raised debate on its potential capability to escape diagnostic assays that target the nucleocapsid protein favoring N gene target failure, whose role is under investigation (48). Furthermore, due to the overlap between the nucleocapsid gene and open reading frame 9b (ORF9b), some nucleocapsid mutations correspond to mutations in ORF9b. This is the case for the P10S and E27-N28-A29 deletion, whose role in modulating viral fitness and pathogenicity is still unknown.

Notably, in 7.7% of Omicron VOC sequences, the ORF8 coding region is completely missing. Although controversial, the ORF8 deletion was reported as early as January 2020 and *in vitro* was associated with higher replication compared to the wild type, suggesting that the lack of this encoding region can provide a potential replicative advantage. This possibility must be confirmed (49).

Finally, other mutations have been detected in the nonstructural proteins encoded by ORF1ab: three new mutations K38R, L1266I, and A1892T and S1265 deletion in NS3 (also known papain-like protease); T492I in NS4 (also found in Delta VOC); P132H in NS5 (main protease); S106-G107-F108 deletion and I189V in NS6; the well-known P323L in NS12 (RNA-dependent RNA polymerase); and lastly I42V in NSP14 (exonuclease responsible for SARS-CoV-2 proofreading activity) (Fig. 1). The impact of these mutations in the main protease and in the polymerase on modulating virological response to the recently approved directly acting antiviral agents (Paxlovid and molnupiravir) deserves further investigation. Preliminary data from Pfizer support the effectiveness of Paxlovid in reducing the risk of hospitalization and death against the Omicron VOC (50). A more recent *in vitro* study has shown that the authorized antivirals remdesivir, molnupiravir and nirmatrelvir (Paxlovid) retain their antiviral activity against the ancestral virus and the VOCs Alpha, Beta, Gamma, Delta, and Omicron (51). Little information is available, with the exception of the NS6 deletion that has been associated with altered SARS-CoV-2 pathogenicity and innate immune evasion (52) and of P323L that has been reported to reduce the binding affinity to remdesivir and to increase the binding affinity of the purine analogues penciclovir and tenofovir (53).

### Conclusions.

The constant process of SARS-CoV-2 genetic diversification has led to the emergence of the newly identified Omicron VOC characterized by a peculiar and unique enrichment of mutations, particularly in the spike glycoprotein and in the RBD, capable of altering SARS-CoV-2 infectivity and antigenicity. The role of these mutations, especially when combined, deserves rapid and further investigation in order to unravel their potential in enhancing viral transmissibility and in jeopardizing the effectiveness of preventive and treatment strategies. The currently available evidence reinforces the need for ongoing molecular surveillance programs to guide the development and usage of vaccines and of therapeutics based on monoclonal antibodies and convalescent-phase sera.

## MATERIALS AND METHODS

In order to fill the above-mentioned gap, 887,475 Omicron B.1.1.529 VOC sequences were retrieved from the GISAID database on 2 February 2022 (39) and used to accurately define the spectrum of mutations in the Omicron VOC (Fig. 1). In particular, sequences were aligned by Bioedit using the NC_045512.2 SARS-Cov-2-Wuhan-Hu-1 isolate as a reference. Mutations were defined as amino acid substitutions/deletions/insertions that occurred in 75% of sequences with respect to the reference. Concerning the number of mutations, we counted each single amino acid variation (point mutation, deletion, or insertion) individually, accordingly to reference (54).

Furthermore, the frequency of currently circulating Omicron VOC mutations was also calculated based on 6,899,523 SARS-CoV-2 sequences from the GISAID database on 2 February 2022. The functional characterization of Omicron spike mutations is presented in Table 1, and residues characterizing the Omicron VOC and the previous four VOCs (Alpha, Beta, Gamma, and Delta) were mapped on the three-dimensional structure of the SARS-CoV-2 spike protein (Fig. 2) using the methodology reported in reference (17).
